# Uncovering the photoexcited dynamics in bis(acyl)phosphine oxide photoinitiators

**DOI:** 10.1039/d5cp01612f

**Published:** 2025-09-12

**Authors:** Marius Navickas, Edvinas Skliutas, Joseph Kölbel, Ricardo J. Fernández-Terán, Mangirdas Malinauskas, Mikas Vengris

**Affiliations:** a Laser Research Centre, Vilnius University Vilnius Saulėtekio ave. 10 LT-10223 Lithuania marius.navickas@ff.vu.lt; b Department of Physical Chemistry, University of Geneva CH-1205 Geneva Switzerland Ricardo.FernandezTeran@unige.ch

## Abstract

Acylphosphine oxide photoinitiators, such as phenylbis(2,4,6-trimethylbenzoyl)phosphine oxide (BAPO) and diphenyl(2,4,6-trimethylbenzoyl)phosphine oxide (TPO), are widely used as sensitisers in UV polymerisation applications due to their favourable properties for UV lithography. However, the underlying photophysical and photochemical mechanisms that govern their performance remain poorly understood. In this study, we systematically investigate these compounds using steady-state and time-resolved fluorescence, as well as electronic and infrared transient absorption spectroscopic techniques. The results show that the lowest electronic excitation of both BAPO and TPO leads to a rapid intersystem crossing (ISC) into the hot triplet manifold—which rapidly generates radicals, further undergoing relaxation and leading to a slower rate of radical formation from the cold triplet manifold. ISC is faster in TPO compared to BAPO, which exhibits more complex dynamics. In addition, time-resolved infrared data reveal that both sensitisers generate radicals (by α-cleavage) on a time scale similar to that of ISC, suggesting that the hot triplet state is very short-lived. Finally, nanosecond-to-microsecond electronic TA spectroscopy revealed multi-stage relaxation of the radicals with the formation of TPO photoproducts remaining detectable on microsecond time scales.

## Abstract 2

Phenylbis(2,4,6-trimethylbenzoyl)phosphine oxide (BAPO) and diphenyl(2,4,6-trimethylbenzoyl)phosphine oxide (TPO) are widely used photosensitizers in UV lithography, exhibiting complex excited-state photodynamics and nonlinear radical recombination.

## Graphical abstract

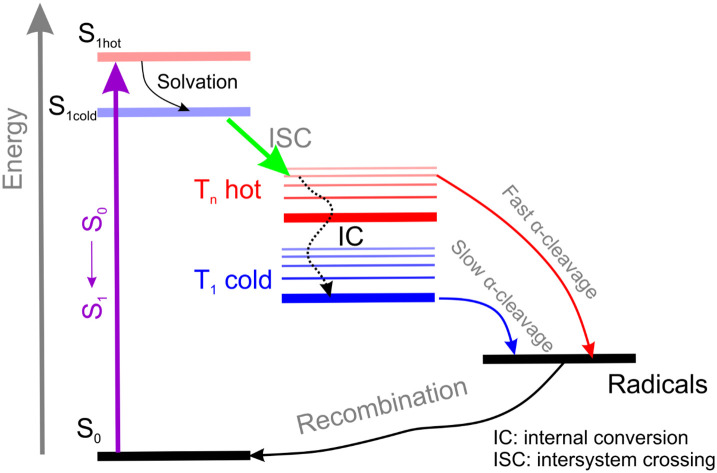

## 1. Introduction

Photopolymerisation has attracted great scientific and technological interest due to a large number of applications, ranging from microelectronics to medical areas.^1–4^ This process enables the manufacturing of complex three-dimensional objects which cannot be cut, assembled, or carved, also at length scales that go beyond conventional machining capabilities.^5,6^

To maintain processing speed, reduce irradiation power and improve the control over the polymerisation process, various photoinitiators can be used.^7,8^ This is especially important in applications where multi-focus or projection-based curing is employed for higher processing throughput. Photoinitiators are essential compounds of photoresists, which are of great interest in a variety of applications such as UV-curable inks, coatings and adhesives, lithography, table-top stereolithography 3D printing, and multiphoton 3D nanoprinting.^9^ These organic molecules absorb light and produce free radicals that trigger the polymerisation reactions.^10^ Since initiation is the first step in the photopolymerisation sequence, it is an important process determining the chemical and physical nature of the final product.

**Fig. 1:**
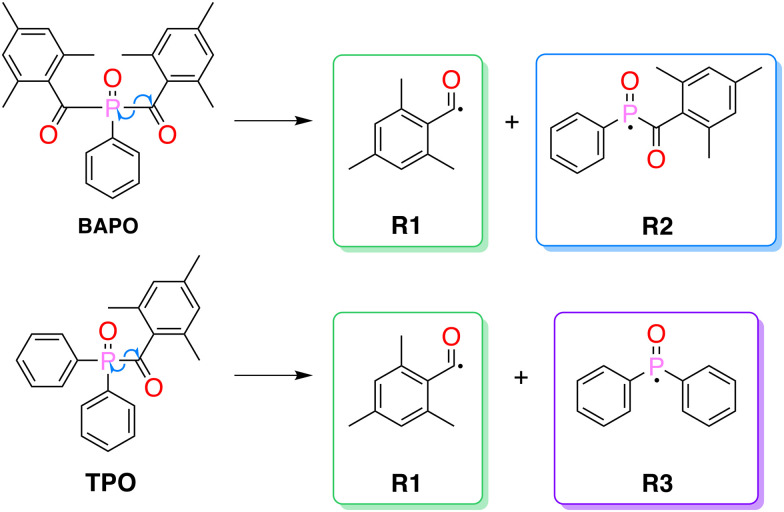
Chemical structures of BAPO and TPO, and their α-cleavage radical photoproducts.

Organic carbonyl-based photoinitiators are often divided into two main categories, based on the mechanism of radical formation: those that undergo a photoinduced α-cleavage (type I) and those that involve hydrogen abstraction by photoexcited molecules (type II).^11^ Type I photoinitiators include acetophenone derivatives, benzoin ethers, aminoketones^12,13^ and acylphosphine oxides.^12,14^ In this work, we will focus on two representatives of the latter group (Fig. 1): phenylbis(2,4,6-trimethylbenzoyl)phosphine oxide (BAPO) and diphenyl(2,4,6-trimethylbenzoyl)phosphine oxide (TPO), which have received considerable recent attention due to presence of different reactive intermediates, and their absorption spectra, which makes them suitable for their use with UV-A and UV/Vis sources.^15^ Photoexcitation of these compounds leads to α-cleavage, generating mesitoyl (R1) and acylphosphinoyl (R2 and R3) radicals.^16,17^

Following the high interest in their applications, Meereis *et al.* have demonstrated that BAPO is remarkably suitable for promoting rapid and efficient polymerisation.^17^BAPO has also become an attractive commercially-available photoinitiator for table-top stereolithography 3D printing,^18^ especially for those employing low-cost LEDs or laser diodes emitting at 405 nm.^19–21^ Multiphoton 3D nanoprinting is one more field, where BAPO is used to modify the photosensitivity of the resins.^22–24^

The initial radicals of BAPO have been extensively studied by different spectroscopic techniques to resolve their structure and decay lifetimes.^16,25^ It was demonstrated that acylphosphinoyl radicals generated by photolysis of acyl and bis(acyl)phosphine oxides show high activity of addition to acrylate prepolymers,^26–29^ illustrating their effectiveness as photoinitiators for free radical polymerisation.

TPO is another compound widely used for photopolymerisation initiation, and is compatible with epoxy acrylate systems^30^ and natural polymers such as lignin^31^ and vinillin.^32^ This photoinitiator belongs to the same bis(acyl)phosphine oxides class, and has a narrow UV/Vis absorption window of 380–425 nm with the maximum peaking at *ca.* 400 nm.^33^ Additionally, TPO exhibits a broader absorption range in the deeper UV region. Exposure of TPO to UV light promotes an α-cleavage photoreaction leading to the formation of R1 and diphenylacylphosphinoyl (Ph_2_P˙ = O) radicals.^34^ An important advantage of TPO and BAPO is their ability to initiate the monomer polymerisation reaction without the assistance of co-initiators.^15^ This can simplify the manufacturing process, and lead to a faster photopolymerisation.^15^ The transient intermediates of TPO have been successfully detected by using both time-resolved and steady-state infrared spectroscopic techniques, which have been used to determine their structures and reactivity.^13,35^

Many studies of reactive intermediates in these photoinitiators focus on the measurements of the reactivity and determination of the polymerisation rate constants, whilst the photophysical mechanisms of initiation have received much less attention. For instance, the primary dynamics of molecule cleavage into radical fragments and the subsequent changes of the absorption spectrum during radical relaxation have been barely investigated. Moreover, the vast majority of radical dynamics studies have relied on laser flash photolysis, with ns–μs time resolution, whilst the ultrafast events of radical formation remain poorly understood. To gain insights into the full dynamic picture covering the fs to μs timescales in these commonly used photoinitiators (BAPO and TPO), we employed steady-state and time-resolved fluorescence, together with ultrafast and fs to μs pump–probe spectroscopies in both UV/Vis and mid-IR spectral regions, supported by density functional theory (DFT) calculations.

## 2. Materials and methods

**Fig. 2:**
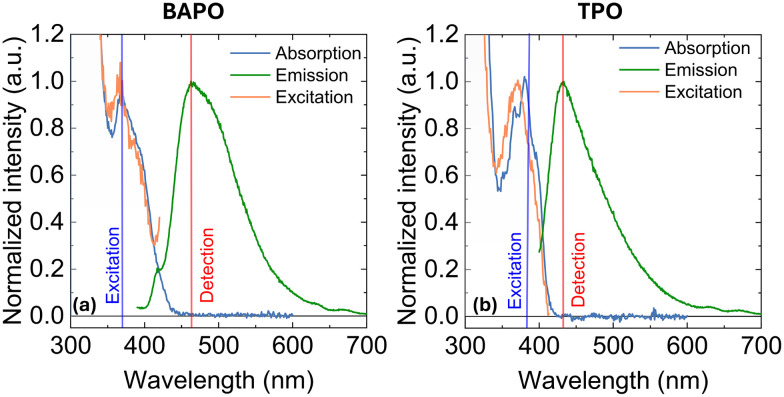
Steady-state absorption, emission and excitation spectra of (a) BAPO and (b) TPO compounds. Vertical lines show the wavelengths used to excite or detect the corresponding emission/excitation spectra.

BAPO and TPO were purchased from Sigma-Aldrich, and used as received. For transient UV/Vis studies, the compounds were dissolved in 2-propanol (Sigma-Aldrich, HPLC Grade), and diluted to an optical density of *ca.* 0.2–0.3 at the lowest absorption band in a 1 mm optical pathlength quartz cuvette (Hellma) as presented in Fig. 2. For time-resolved infrared (TRIR) studies, the compounds were dissolved in dichloromethane (Sigma-Aldrich, HPLC grade). Also, the comparison UV/Vis TA experiments in dichloromethane were performed, and the obtained datasets for both BAPO and TPO are presented in the SI (Fig. S1 and S2).

Steady-state absorption and emission/excitation spectra of the 2-propanol solutions were recorded using a Shimadzu UV-3101PC UV/Vis absorption spectrometer, and a PerkinElmer LS55 fluorescence spectrometer, respectively. For steady-state fluorescence measurements, the sample solutions were diluted to an optical density below 0.1 in a 1 × 1 cm Hellma quartz cells.

Time-resolved emission spectra were collected at different wavelengths using a time-correlated single-photon counting system (TCSPC), integrated in the Harpia-TF time-resolved fluorescence spectrometer (Light Conversion Ltd). In these experiments, the third harmonic (344 nm) of an Yb:KGW laser (Pharos, Light conversion, Ltd) operating at 1030 nm, and delivering 200 fs pulses at 100 kHz repetition rate. The fluorescence excitation power was set to *ca.* 65 μW at 100 kHz pulse repetition rate.

Ultrafast transient UV/Vis absorption (TA) spectra were measured using a Harpia-TA (Light Conversion, Ltd) commercial transient absorption spectrometer. The pump pulse was generated by an optical parametric amplifier (TOPAS-800, Light Conversion) which was pumped by a commercial Ti:Sa femtosecond laser (Coherent Libra; delivering 800 nm, 50 fs, 2.5 W pulses at 1 kHz). For ultrafast pump–probe experiments, the BAPO and TPO samples were excited at the lowest absorption band. The excitation power for all performed experiments was tuned to vary by an order of 2% of the excited states, to avoid any multiphoton ionization effects. Detailed values of absorbed photons per pulse as well as concentrations of the excited states can be found in Table S1 of SI. To probe the population dynamics, we used a white light supercontinuum, generated in a mechanically-translated CaF_2_ plate. The polarisation of the pump beam was set to magic angle (54.7°) respect to the probe beam. The pump–probe delay was controlled with an optical delay line, in the range from fs to a few ns. The TA spectra were recorded in a 1 mm pathlength Hellma quartz flow cell to ensure that each laser pulse irradiated a fresh volume of sample. At least, 10 scans with 10 bathes of 250 shots for each TA spectra were recorded and averaged over the number of performed scans that were virtually identical.

To extend the probe time window beyond the range of the optical delay line, an additional pump laser (NL640, Ekspla), which emitted 3 ns pulses at 355 nm, was used as an alternative excitation source. The samples were excited with 1 μJ pulses and 120 μm spot size beam. The operation of this laser was electronically synchronised with the Ti:Sapphire laser using the available digital outputs of the SDG-Elite synchronisation and timing unit (part of the Libra laser system). Using this arrangement, the ns laser pulse could be timed to arrive between 800 μs before to 50 ns after the femtosecond probe pulse, with a precision of 250 ps.

Time-resolved infrared spectra (TRIR) were collected at the University of Geneva. In brief, the output of a Ti:Sa amplifier (Spectra-Physics, Solstice ACE) producing *ca.* 100 fs pulses centred at 800 nm was used to pump two TOPAS-C optical parametric amplifiers (Light Conversion, Ltd). The output of the first TOPAS-C was fed into a non-collinear difference frequency generation (NDFG) stage with a AgGaS_2_ crystal, producing mid-IR pulses centred at 1600 or 1800 cm^−1^, with a FWHM of *ca.* 170 cm^−1^. The second TOPAS-C was used to produce 640 nm pulses, which were then frequency doubled in a bismuth borate (BiBO) crystal, producing UV excitation pulses at 320 nm. The 400 nm pulses used in complementary TRIR experiments were obtained by frequency-doubling a portion of the fundamental 800 nm light in a β-barium oxide (BBO) crystal. The fundamental light was removed in both cases by the use of a Glan–Taylor polariser, after which an achromatic *λ*/2 waveplate was used to rotate the polarisation to magic angle (54.7°) relative to the s-polarised probe beam. The pump–probe delay was set with the help of a mechanical delay stage. The mid-IR beam was split into probe and reference beams using an uncoated CaF_2_ wedge, and both beams were passed through two wire grid polarisers—to clean the polarisation and attenuate, respectively—both the probe and reference beams. The two mid-IR beams were focussed at the sample to a spot size of *ca.* 120 μm using off-axis parabolic mirrors, and the pump was focussed with the help of a lens (*f* = 75 cm), to a spot size of *ca.* 240 μm. All other experimental parameters for each spectroscopic technique are summarised in a Table S2 in the SI.

After the sample, both probe and reference were imaged into a spectrograph and dispersed with a 150 l mm^−1^ grating into a 64 × 2 pixel liquid-nitrogen cooled HgCdTe detector. A multichannel referencing scheme, as described by Ge and co-workers was used to account for fluctuations in the probe beam.^36,37^ The resulting TRIR spectra were obtained after averaging at least 4 scans, with 4 batches of 500 shots at each time delay. The samples were recirculated in home-made cells with 2 mm thick CaF_2_ windows and a 400 μm PTFE spacers, and the scans were checked for scan-to-scan stability of the observed signals. Note that some spectral artifacts are observed due to the TRIR measurements being made in the absence of suitable purging, and contain the imprinted signatures of water vapour and CO_2_, evident as sharp features (especially in the 1550–1600 cm^−1^ region).

Density functional theory (DFT) calculations were performed in Gaussian 16, rev. A.03,^38^ at the B3LYP/6-311++g(d,p) level of theory,^39–41^ including the effects of the DCM solvent using the IEFPCM model.^42^ All structures were optimised under tight convergence criteria, and were found to be true minima in their corresponding potential energy surfaces by the absence of negative (imaginary) harmonic frequencies. Difference mid-IR spectra were obtained by convolution with a Lorentzian line shape function of 8 or 10 cm^−1^ FWHM for the ground-state and excited-state/photoproduct spectra, respectively. The harmonic frequencies were scaled by 0.983 to better match the experimentally obtained values.

Global/target analysis was performed on both time-resolved fluorescence and pump–probe (UV/Vis and mid-IR) data, to extract meaningful time constants and species- or evolution-associated spectra,^43–45^ using the CarpetView software (Light Conversion, Ltd).

## 3. Results

### 3.1. Steady-state and time-resolved emission spectroscopy

We begin by analysing the steady-state absorption and emission spectra of BAPO and TPO in 2-propanol solutions (Fig. 2). The absorption spectra (solid blue lines) of both BAPO and TPO extends to around 420 nm, with absorption maxima at *ca.* 360 nm and 380 nm, for BAPO and TPO, respectively. Their emission spectra (solid green lines) consist of a broad, structureless band spanning the range from 420–600 nm, with emission maxima at *ca.* 470 nm and 430 nm for BAPO and TPO, respectively. Their excitation spectra (solid orange lines) closely match the corresponding absorption profiles, indeed showing that the observed emission is a result of direct excitation.

**Fig. 3:**
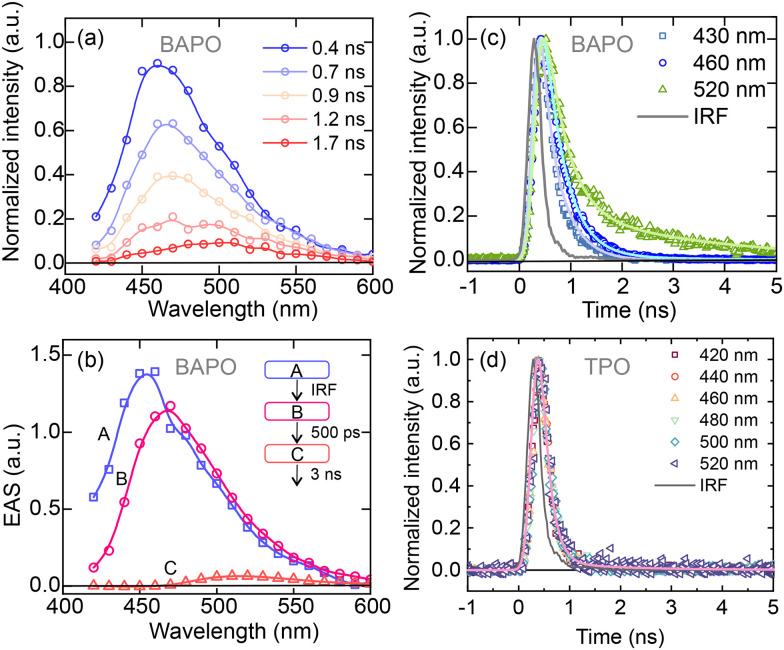
(a) Time-resolved emission spectra of BAPO in 2-propanol. (b) Evolution-associated spectra retrieved from global analysis. Panels (c) and (d) represent, respectively, the fluorescence decay traces of BAPO and TPO in 2-propanol at different detection wavelengths.

Time-resolved emission spectra (Fig. 3), obtained after pulsed excitation of BAPO at 344 nm, show a continuous red shift from 470 nm to 520 nm (*cf.* 400 ps *vs*. 1.7 ns spectra in Fig. 3a), accompanied with a decay that is almost complete within 1 ns. The evolution-associated spectra (EAS) obtained from global analysis reveal an IRF-limited component (the FWHM of the IRF is 300 ps), and two time constants of *ca.* 500 ps and 3 ns—the latter resulting in a significant decrease in intensity. Inspection of the emission decay traces at different detection wavelengths (Fig. 3c), reveals slightly longer decay times in the red emission wavelengths, suggesting non-trivial relaxation dynamics in the excited state. In contrast, the corresponding time-resolved emission data of TPO (kinetic traces shown in Fig. 3d) show no emission wavelength dependence, and all kinetic traces decay with an IRF-limited time constant.

### 3.2. Transient electronic spectroscopy

**Fig. 4:**
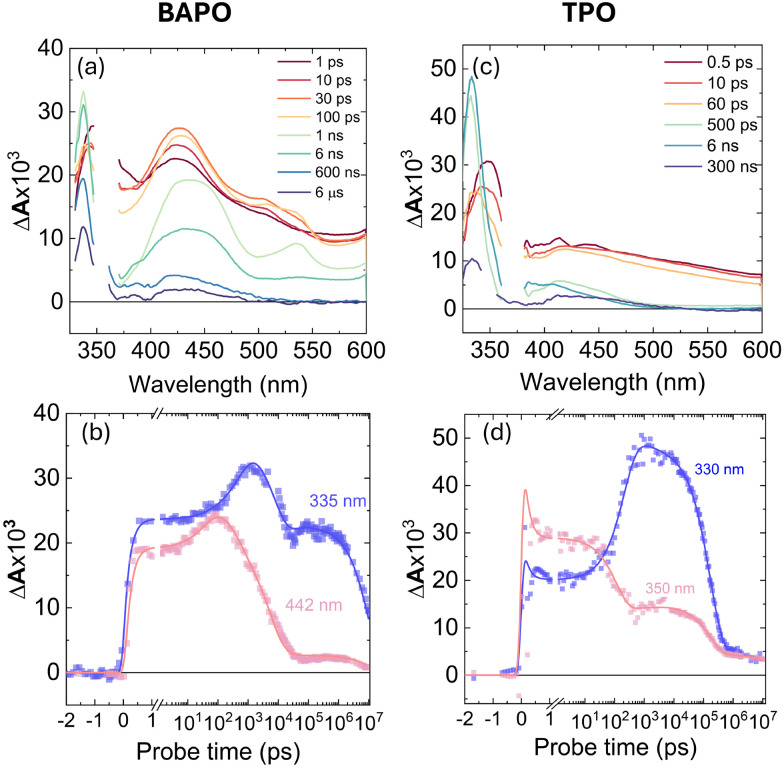
TA spectra (top row) and selected kinetic traces (bottom row) obtained after excitation with 360 nm pulses (fs–ns) or 355 nm (ns–μs), of solutions of BAPO (a and b) and TPO (c and d), respectively. The spectra were merged at a delay of 6 ns.

To gain further insight into the excited-state photodynamics of BAPO and TPO upon UV excitation, we now turn to transient UV/Vis absorption spectroscopy (TA). In these experiments, we combined broadband fs–ns and ns–μs pump–probe techniques to probe the entire range of timescales needed to fully address the complex photochemistry of these compounds. The resulting spectra and selected kinetic traces are shown in Fig. 4.

As shown in Fig. 4a, within several picoseconds after excitation of BAPO, we observe a broad and featureless excited-state absorption (ESA) background, with distinct spectral maxima around 335 nm and 442 nm which further develop over time. The peaks around 335 nm and 442 nm become more pronounced, and after approximately 30 ps, an additional peak starts to form at *ca.* 530 nm. The spectral feature around 335 nm does not change significantly after *ca.* 1 ns.

An interesting spectral transformation also takes place on a ns–μs timescale. The decrease of ESA at 335 nm is slower compared to the ESA at 442 nm, and the IA feature at 530 nm becomes negligible after *ca.* 6 ns. Kinetic traces at 335 nm and 442 nm (Fig. 4b) reveal the temporal evolution of the BAPO transients. The 335 nm signal decays rapidly within picoseconds, whilst the 442 nm signal remains stable. Both traces then grow, with the 442 nm signal peaking at 200 ps and the 335 nm signal continuing to rise until *ca.* 1 ns. Subsequently, both signals decay, with the 442 nm trace showing a more gradual decline compared to the steeper slope of the 335 nm trace. Both traces plateau at *ca.* 20 ns, followed by a slow decay over *ca.* 10 μs. A residual TA signal persists, attributed to long-lived radical species responsible for photoinitiator activity.

For comparison, the photoinduced dynamics of TPO in 2-propanol were also studied. Excitation at 360 nm produces a broad IA band peaking at *ca.* 330 nm, and a weaker peak around *ca.* 425 nm (Fig. 4c). Kinetic traces at 330 nm and 350 nm (Fig. 4d) reveal more intricate sub-picosecond dynamics: the 335 nm signal initially decreases slightly, remains stable until *ca.* 30 ps, then grows to a maximum at *ca.* 800 ps. Meanwhile, the 350 nm signal remains stable until *ca.* 30 ps before decaying to a plateau, coinciding with the 330 nm peak. Both traces exhibit multi-stage relaxation over 1–100 ns, with weaker residual signals compared to BAPO, and notably, the lack of an ESA feature around 530 nm in the TPO spectra.

### 3.3. Quantum chemical calculations

**Fig. 5:**
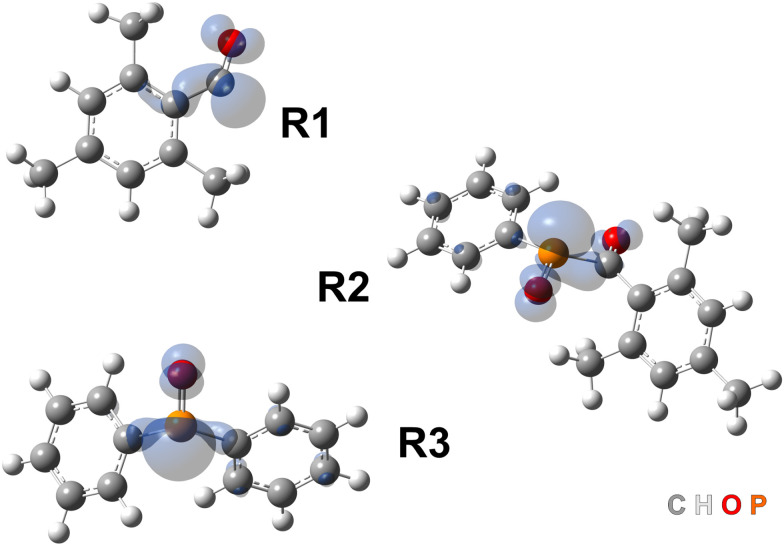
Optimised structures and spin density isosurfaces of the mesitoyl (R1) and acylphosphinoyl (R2 and R3) radicals relevant for this study. Isosurfaces plotted at 0.005 a.u. The element colour code is included in the figure (bottom right corner).

Density functional theory (DFT) calculations reveal interesting insights into the photochemical reaction and the properties of the obtained photoproducts. Optimisation of both BAPO and TPO in the lowest singlet excited state (S_1_) yielded stable structures, whilst the optimisation of BAPO in the lowest triplet excited state (T_1_) resulted in cleavage of the C–P bond, which was not the case for TPO. The expected mesitoyl (R1) and acylphosphinoyl radicals (R2 and R3) were also optimised at the same level of theory in their doublet spin configuration, and the obtained structures and spin density isosurfaces are shown in Fig. 5. The *XYZ* coordinates of all relevant structures are provided in the SI.

As is evidenced from the plots in Fig. 5, the spin density is largely localised in the C=O or P=O moieties for R1 and R2/R3, respectively.

We used the results of these calculations, and the corresponding harmonic vibrational spectra, to guide our interpretation of the experimental time-resolved infrared spectra, discussed next.

### 3.4. Time-resolved infrared spectroscopy

Transient infrared spectra (TRIR) of BAPO and TPO in the C=C and C=O stretching region (1550–1660 cm^−1^ and 1740–1875 cm^−1^) were recorded upon excitation at both 400 nm and 320 nm. We used dichloromethane (DCM) as solvent for these experiments due to its higher IR transparency relative to 2-propanol.

**Fig. 6:**
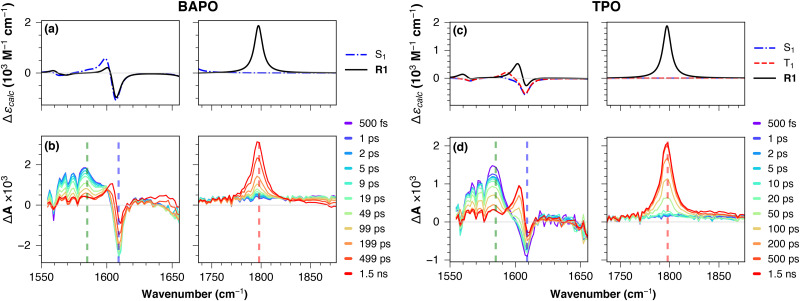
Calculated mid-IR difference spectra (top row) and experimental TRIR spectra following 400 nm excitation in DCM (bottom row), of BAPO (a) and (b) and TPO (c) and (d). Vertical dashed lines indicate the probe wavelengths used for the kinetic cuts shown in Fig. 7, corresponding to the bleach of the parent compound (1609 cm^−1^, blue), the transient associated with the S_1_ state (1585 cm^−1^, green), and the transient of the photoproduct acyl radical band (1798 cm^−1^, red). The calculated frequencies were scaled by 0.983 to better match the experiment. Optimisation of BAPO in the T_1_ state led to breaking of the C–P bond, suggesting a dissociative behaviour in this state (see text for discussion).

Herein, we will focus on the results obtained with 400 nm excitation. The complementary datasets associated with 320 nm excitation are shown in the SI. The TRIR data for both compounds is presented in Fig. 6. The earliest time spectrum (500 fs) shows, in both cases, a negative signal at 1609 cm^−1^, corresponding to the ground-state bleach (GSB) of the C=C ring stretching modes localised on the mesityl moiety.

In both cases, we also observe a prompt ESA band around 1585 cm^−1^, corresponding to the C=C ring stretching modes localised on the Ar_2_P=O moieties of excited BAPO and TPO in the S_1_ state. These signals do not show any significant spectral evolution, decaying in *ca.* 100 ps, and giving rise to a weak transient at *ca.* 1605 cm^−1^ (attributed to the C=C ring stretching mode of the mesitoyl radical, R1) and a strong transient at *ca.* 1798 cm^−1^ (attributed to the C=O stretching mode of R1). The calculated mid-IR difference spectra (Fig. 6, panels a and c) reproduce almost quantitatively the experimental data, supporting both the level of theory and the reactivity scheme used to interpret our results.

**Fig. 7:**
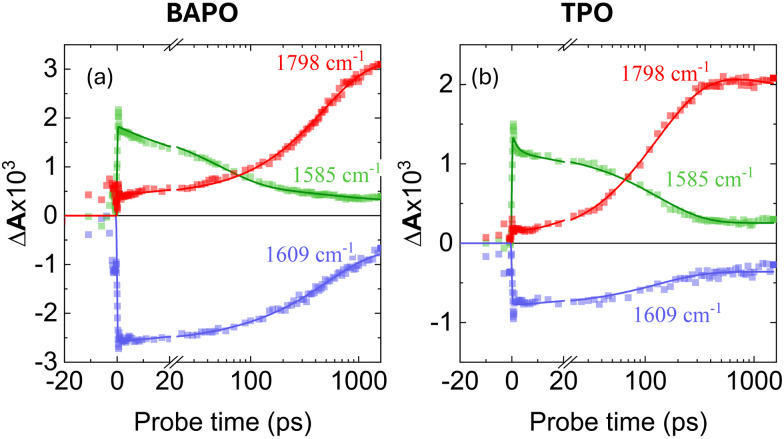
Kinetic traces corresponding to the bleach of the parent compound (1609 cm^−1^, blue), the transient associated with the S_1_ state (1585 cm^−1^, green), and the transient of the photoproduct mesitoyl radical band (1798 cm^−1^, red), obtained from the TRIR data of BAPO (a) and TPO (b) upon photoexcitation at 400 nm. Symbols indicate measured data, and solid lines indicate the results from the global fits.

A closer inspection of kinetic traces at selected positions (Fig. 7), corresponding respectively to the bleach of the parent compound (1609 cm^−1^, blue), the transient associated with the S_1_ state (1585 cm^−1^, green), and the transient associated with the C=O stretching of the mesitoyl radical photoproduct (1798 cm^−1^, red), the latter in excellent agreement with previous TRIR reports by George and co-workers.^13^

The 1609 cm^−1^ trace exhibits a bi-exponential recovery with a fast component of 500 ps, but does not fully recover. This fast component well-matches to the emission lifetime (the detailed fitting is presented in Fig. S5). The agreement between the BAPO emission lifetime and the GSB recovery time constant indicates that the observed emission arises from S_1_ → S_0_ relaxation. Concurrently, the 1585 cm^−1^ trace shows a sub-nanosecond decay, followed by the gradual rise of a new feature at 1798 cm^−1^. The decay of the 1585 cm^−1^ band and the rise of the 1798 cm^−1^ band occur on the same timescale. The lack of GSB recovery until the new ESA appears suggests that most of the BAPO excited-state population undergo decomposition, without repopulating S_0_.

We obtained similar results for TPO under these conditions (*λ*_exc_ = 400 nm, DCM), as expected from their similarity. Although its TRIR dynamics have been previously discussed,^46^ they exhibit spectral features analogous to BAPO, making TPO a suitable reference. The kinetic traces of TPO mirror those of BAPO: the ESA at 1585 cm^−1^ decays, whilst the ESA at 1798 cm^−1^ grows on the same timescale. Notably, the 1609 cm^−1^ GSB kinetics show a bi-exponential recovery with time constants *τ*_1_ = 130(10) ps (for BAPO, it is 500(50) ps) and an infinite component (*τ*_2_), indicating dynamics taking place beyond the *ca.* 1.6 ns timescale accessible with our optical delay line, as expected from the photochemistry of these compounds and from ns–μs TA experiments.

The short recovery time of the GSB and the short emission lifetime in BAPO and TPO indicate a quick decay of the singlet state. This short lifetime of the excited state may result from direct cleavage or intersystem crossing, which leads to the population of triplet states. Altogether, the TRIR results confirm the identity of the photoproducts (R1, R2 and R3), as well as the lack of direct α-cleavage from the singlet excited state. This is in line with previous reports from Turro and co-workers,^47^ who state that α-cleavage in cyclic ketones is expected to be *ca.* 100 times faster from the triplet excited state than from the singlet excited state.

### 3.5. Naphthalene quenching experiments

**Fig. 8:**
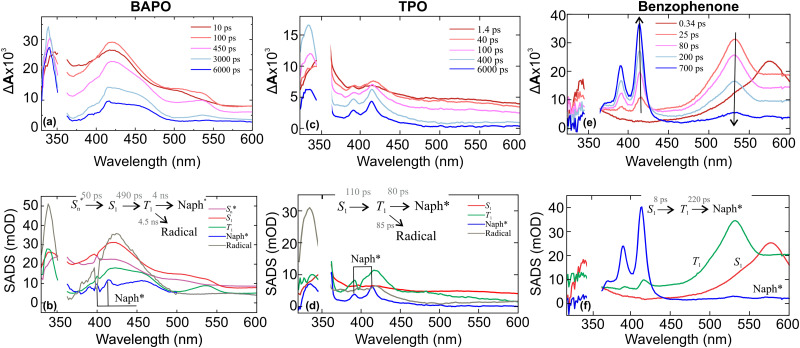
(a), (c) and (e) TA spectra of BAPO, TPO and benzophenone 2-propanol solutions, containing naphthalene and (b), (d) and (f) SADS, obtained from global analysis. The empty region in spectra indicate area omitted due to the scattering of the pump pulse. The concentrations of BAPO, TPO and naphthalene were 3 mM, 6 mM and 300 mM, respectively.

To test the involvement of the triplet state, and to confirm its lifetime, we performed ultrafast UV/Vis pump–probe experiments (*λ*_exc_ = 350 nm) aiming for triplet sensitisation, in 2-propanol solutions of BAPO and TPO, with naphthalene as triplet sensitiser. Since triplet energy transfer requires orbital overlap (*i.e.* direct contact of the molecules), we use quite high naphthalene concentrations of 300 mM. The main results from these experiments are summarised in Fig. 8.

In Fig. 8a and b we present the results of triplet sensitisation of BAPO and TPO, respectively. As observed, for BAPO the TA spectra on the picosecond time scale are not influenced by naphthalene. Only at *ca.* 450 ps some naphthalene triplet signatures (fine peaks at 390 and 415 nm)^48^ start to appear and become more visible after *ca.* 3 ns. For TPO, the triplet energy transfer seems to be faster, and naphthalene triplet features are better resolved.

To confirm triplet–triplet energy transfer in naphthalene solutions, we measured TA dynamics of benzophenone with 500 mM naphthalene (Fig. 8e), since benzophenone has a very high triplet quantum yield. Then, if the appearance of peaks at 390 nm and 415 nm arises from naphthalene triplet state absorption, it should be strongly prominent in the benzophenone-naphthalene solution. Indeed, the same characteristic naphthalene triplet state absorption appears and increases over several hundred picoseconds; meanwhile, the absorption of benzophenone singlet state at 530 nm decreases. That indicates a fast triplet–triplet energy transfer between benzophenone and naphthalene.

## 4. Discussion

The current understanding of BAPO and TPO photochemistry states that triplets are usually precursors of radicals. This hypothesis is widely supported in the field of ESR spectroscopy,^16,25^ however, the actual time constants for intersystem crossing (ISC) and subsequent α-cleavage have not been clearly resolved. To determine the time scales of triplet state population in BAPO and TPO, we start with naphthalene quenching experiments. To analyze the femtosecond-to-nanosecond UV/Vis TA dataset of BAPO and TPO solutions containing naphthalene as a triplet sensitizer, we apply connectivity schemes to fit the data, which are also illustrated in the insets of Fig. 8. In the analysis of the BAPO TA dataset, the pathway S_1_ → T_1_ corresponds to an ISC occurring within 490 ps, while the transition T_1_ → Naph evolves over 4.0 ns, representing the triplet–triplet energy transfer between BAPO and naphthalene. Furthermore, the transition 
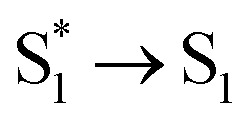
 denotes the relaxation of the excited singlet state. The species-associated difference spectrum (SADS) of Naph reveals characteristic ESA features at 390 nm and 415 nm, which confirm the presence of triplet states in BAPO photodynamics. In case of TPO, these triplet features appear even faster, within 80 ps, and are better resolved. Although the triplets in BAPO and TPO that are surrounded by naphthalene undergo quenching, a fraction of them still undergo α-cleavage, resulting in the formation of free radicals. This process is illustrated by a pathway T_1_ → radicals, with a corresponding time constant of 4.5 ns. Despite the findings, there is a puzzling aspect regarding the timescale of triplet energy transfer for BAPO and TPO. In case of BAPO, the energy transfer to naphthalene takes 4.5 ns, whereas for TPO, it only takes 80 ps. Since the triplet sensitizer in both cases is the same and BAPO together with TPO have similar structures, we would expect the energy transfer time constants to be comparable.

The establishment of the role of the triplets in both molecules implies that fast emission decay might be related to ISC; in other words, the singlet state in both molecules is deactivated due to the population of triplets. Since the line shape of the absorption and excitation spectra are similar, we conclude that the emissive state is produced with the same probability from the absorbing state. At the same time, the fact that the Stokes shift is in the order of thousands of cm^−1^, and the spectrum is broad, points to complex relaxation taking place in the excited state. Of particular interest here is the emission decay of BAPO. Here, after 500 ps, we obtain a small residual signal, spreading around 520 nm and surviving nanoseconds. Given the involvement of the triplet state from naphthalene experiments, we hypothesized that this residual signal could be phosphorescence or delayed fluorescence. This hypothesis became an argument when we measured the emission decay kinetics in oxygen-free BAPO solution. We found a slight prolongation of emission lifetime (see SI, Fig. S6). This effect was reversed upon bubbling the solution once more with air, confirming that the obtained changes are related to the content of oxygen in the solution. This is indeed strong evidence to attribute the red emission decaying in nanoseconds to phosphorescence of the triplets.

**Fig. 9:**
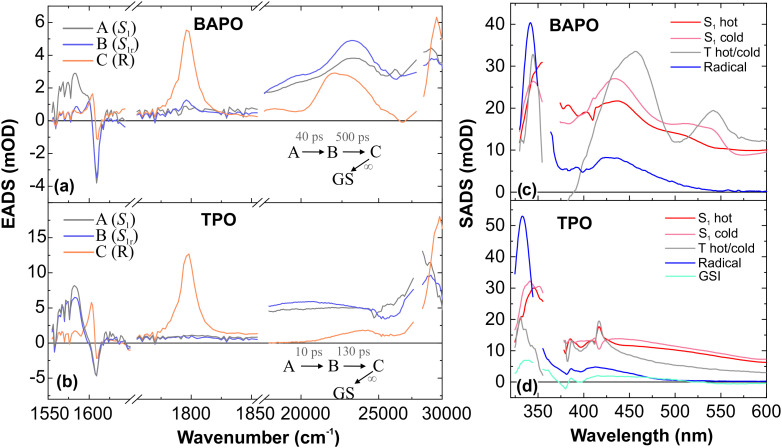
Evolution-associated difference absorption spectra (EADS) and time constants obtained from a global analysis of the (a) BAPO and (b) TPO TRIR-UV/Vis data. The panels (c) and (d) show the SADS of femtosecond-microsecond UV/Vis BAPO and TPO TA data obtained by assuming the model in Fig. 10. The TRIR spectra in panel (a) and (b) were scaled to match their intensities with UV/Vis TA spectra.

Assuming that triplets are precursors of radicals, they should emerge over nanoseconds for BAPO, but TRIR shows different. The transition B → C (see Fig. 9a) with a lifetime close to that of ISC shows an appearance of characteristic frequencies of C=O stretching in Mes-CO radical.^46^ Furthermore, the transition in the IR region is also accompanied by a change in UV/Vis, but the characteristic EADS C of UV/Vis TA does not resemble the spectrum of the radical; it may rather be attributed to the triplet features. This contradicts either naphthalene quenching and emission results that suggest ten times slower development of the radicals than obtained by TRIR. However, these experimental results seem to contradict each other only under the assumption that the photodynamics in BAPO and TPO are sequential, *i.e.*, singlet → triplet → radicals. The rapid formation of radicals and a small fraction of triplets detectable on a nanosecond timescale points to a branched reaction scheme of both molecules.

The rapid formation of radicals can occur from either singlet or triplet states. However, based on TRIR measurements and in agreement with Turro and co-workers,^47^ we observed this process occurring on a timescale similar to ISC, therefore, it is more likely that radicals are produced from triplet states. During the radical formation stage, two types of triplet states must be involved: hot and cold triplets. Hot triplets possess much excess energy and either quickly generate free radicals or undergo internal conversion to a lower energy state, producing cold triplets, where α-cleavage slows down. Additionally, molecules being in this relaxed triplet configuration can further replenish the ground state (S_0_) by emitting photons detectable in the nanosecond time scale. All considered processes, especially those that cover longer timescales, can be resolved in UV/Vis TA data.

**Fig. 10:**
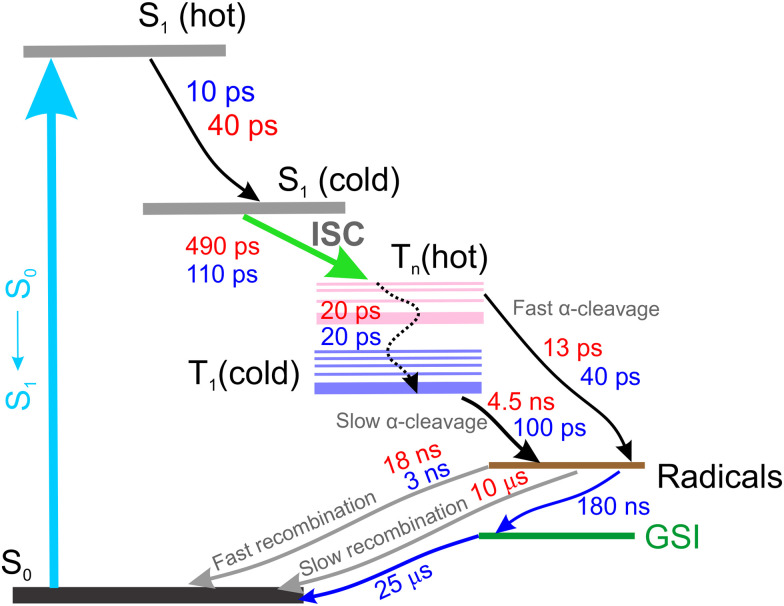
Reaction scheme, summarizing the mechanisms and their timescales in BAPO (red) and TPO (blue). The GSI component represents a small fraction of the TPO photoproduct, detectable on a microsecond time scale.

To account for possible branching in the excited-state kinetics, we used a target model, presented in Fig. 10. The model nicely fits the kinetic traces (Fig. 4b and d) and yields SADS, which are shown in Fig. 9c and d. The first two components S_1_(hot) and S_1_(cold) denote either vibrational relaxation and solvation of the excited singlet state and both SADS are nearly similar. Furthermore, the EADS of TRIR obtained on the same timescale (EADS A and B) have the same vibrational frequencies. However, EADS B exhibits only loss of the ESA, suggesting that the observed changes belong to the same excited state. Of particular importance in this reaction scheme is ISC (S_1_(cold) → T_*n*_(hot)), producing hot and cold triplets that further boost photofragmentation. In the TRIR data we do not observe significant changes attributed to this process. However, UV/Vis data show some spectral changes when going from singlet to triplet electronic configuration. This can be seen when comparing the UV/Vis EADS S_1_(cold) and T_*n*_(hot). After an ISC, 2/3 of the hot triplet state population directly produces mesitoyl and acylphosphinoyl radicals,^26,35,49^ yielding the EADS ‘Radicals’, which displays the features of the previously measured acylphosphinoyl radical spectrum.^16^ The photofragmentation is accompanied by internal conversion of the hot triplet, T_*n*_(hot) → T_1_(cold), within 20 ps into a more stable triplet configuration, T_1_(cold), yielding the discussed radical pairs within 4.5 ns. The same cold triplet was detected by measuring the emission dynamics. For TPO, the branching ratio of internal conversion to initial radical formation is slightly different (1 : 2). On the other hand, the TRIR shows radical formation within 130 ps. Assuming such a branching ratio, the radical features are thus expected on a similar time scale. The plausibility of the model could also be confirmed by concentration plots of the species, presented in Fig. S7. When we compare the growth of TRIR ESA at 1798 cm^−1^ and the superposition of T_*n*_(hot) and ‘Radicals’ species temporal population profiles, we obtain a perfect overlap case of BAPO. For TPO, UV/Vis TA temporal profile *c*(*t*) superposition of the latter species and 1798 cm^−1^ TRIR ESA just grow at similar rates. TCSPC kinetics of BAPO at 520 nm can also be reconstructed in the same way as shown in Fig. S8, confirming the agreement between UV/Vis TA and TCSPC measurements. Coming back to naphthalene quenching of BAPO triplets, now assuming the presence of hot triplets, the BAPO-naphthalene quenching time constant seems to be plausible – once the triplets are formed, they are deactivated by a rapid α-cleavage and only the cold triplets, where the radical splitting is slower, can undergo energy transfer to naphthalene.

The kinetic traces in Fig. 4b and d indicate that recombination of radicals in both molecules seems to be complex and occurs over several steps; therefore, to simplify this process in the global analysis, we use several parallel decays from the ‘Radical’ species at different time constants. Initially, the number of radicals is high, so the probability of their mutual collisions at the beginning is highest and leads to a rapid recombination. In other words, at first the radicals recombine quickly due to frequent collisions. As the concentration of radicals decreases, collisions become less frequent, and recombination slows down.^50^

The global analysis shows that the slow recombination of BAPO radicals takes about 10 μs (Radicals → S_0_) while the fast occurs in just over 18 ns. In TPO, the fast recombination is even faster – 3 ns. For TPO, we use an additional component (GSI) to account for slowly decaying transients. The latter component represents a slowly decaying photoproduct of TPO radicals, which is formed within 180 ns and relaxes with a 25 μs time constant. It is important to note that a quantitative description of radical decay kinetics cannot be achieved using a single-exponential model only. This is because the actual decay rate depends on the concentration of the produced radicals. Consequently, our kinetic model can only be used to qualitatively estimate the timescale of the relaxation process under our experimental conditions.

## 5. Conclusions

Ultrafast transient absorption measurements on commonly used phosphine oxide photoinitiators BAPO and TPO, with the absence and presence of naphthalene in solutions, have shown that these molecules undergo ultrafast intersystem crossing (490 ps in BAPO and 110 ps in TPO), followed by α-cleavage, creating chemically active acylphosphinoyl radical species. Moreover, time-resolved IR spectroscopy revealed that radicals in both compounds start to form quickly, on a similar time scale as intersystem crossing, indicating that ISC is a primary factor influencing the activity of such compounds. In solution, these radicals were found to recombine in a multi-stage fashion, with a fraction of the radical photoproducts (TPO) remaining detectable on a microsecond time scale. A detailed picture of the observed photodynamics (Fig. 10) was constructed in terms of the global analysis kinetic model, providing quantitative inputs for further analysis of the dynamics of photopolymerisation reactions. Observations on BAPO and TPO clearly show the involvement of triplet states which are hypothesized to be precursors of the radicals important in photoinitiation. If the hypothesis is correct, this would imply that the development of compounds with higher intersystem crossing rates may result in more efficient photoinitiators for radical photopolymerisation.

## Conflicts of interest

There are no conflicts to declare.

## Supplementary Material

CP-027-D5CP01612F-s001

CP-027-D5CP01612F-s002

## Data Availability

The data that support the findings of this study are available in the supplementary material (SI) of this article. See DOI: https://doi.org/10.1039/d5cp01612f
