# Supplementary material for: Uncovering the photoexcited dynamics in bis(acyl)phosphine oxide photoinitiators
Source: Phys Chem Chem Phys. 2025 Sep 12;27(38):20592–601. doi: 10.1039/d5cp01612f (PMC12427173; doi:10.1039/d5cp01612f)
Supplement: CP-027-D5CP01612F-s002 [file CP-027-D5CP01612F-s002.pdf]

Supplementary Information (SI) for Physical Chemistry Chemical Physics.
This journal is © the Owner Societies 2025

Supplementary information

Uncovering the photoexcited dynamics in bis(acyl)phosphine oxide photoinitiators

Marius Navickas,[a] Edvinas Skliutas,[a] Joseph Kölbel,[b] Ricadro J. Fernández-Terán,[b] Mangirdas Malinauskas[a] and Mikas Vengris[a]

[a]Vilnius University, Laser Research Center, Saulėteko av. 10, LT-10223 Vilnius, Lithuania; E-mail: marius.navickas@ff.vu.lt

[b]Department of Physical Chemistry, University of Geneva, CH-1205 Geneva, Switzerland; E-mail: Ricardo.FernandezTeran@unige.ch

Table S1. Detailed summary of the experimental parameters used for each technique and comparison of chromophore concentration vs. concentration of absorbed photons per pulse.

**Technique**	**Experimental parameters**	**Result**
UV/Vis TA	Excitation wavelength (nm): 360	Absorbed photons in the probed volume: 7.5x10^{10} (6.86x10^{10})
	Excitation beam diameter (um): 150	
	Probe beam diameter (um): 50	
	Optical density (OD): 0.34 (0.3)	Chromophores in probed volume: 3.5x10^{12} (7x10^{12})
	Concentration of chromophores (mM): 3 (6)	
	Excitation power (uW): 330	
	Pulse repetition rate (Hz): 483	**Concentration of absorbed photons: 63 µM (58 µM)** **Percentage of the excited molecules:~2% (~1%)**
	Cuvette pathlength (mm): 1	
TRIR	Excitation wavelength (nm): 400	Absorbed photons in probed volume: 2.79x10^{11} (2.79x10^{11})
	Excitation beam diameter (um): 240	
	Probe beam diameter (um): 120	
	Optical density (OD): 0.8 (0.8)	Chromophores in the probed volume: 1.6x10^{13} (4.63x10^{13})
	Concentration of chromophores (mM): 6(17)	
	Excitation power (uW): 330	
	Pulse repetition rate (Hz): 500	**Concentration of absorbed photons: 93.2 µM (93.2 µM)** **Percentage of the excited molecules: ~1.6% (~0.55 %)**
	Cuvette pathlength (mm): 0.4	
TCSPC	Excitation wavelength (nm): 344	Absorbed photons in the probed volume: ~1.92x10^{9} (~1.92x10^{9})
	Excitation beam diameter (um): 150	
	—	
	Optical density (OD): 0.2 (0.2)	Chromophores in the probed volume: 1.7x10^{13} (4.5x10^{13})
	Concentration of chromophores (mM): 1.6 (4.2)	
	Excitation power (uW): 65	
	Pulse repetition rate (kHz): 100	**Concentration of absorbed photons: 0.039 µM (0.039 µM)** **Percentage of the excited molecules: 0.002% (0.00 1 %)**
	Cuvette pathlength (mm): 1	

Table S2. Summary of the time resolution, the concentration of absorbed photons per pulse and the chromophore concentration, determined for each spectroscopic technique. The numbers in parentheses represent the values for TPO

Technique	λ_{ex} (nm)	τ (ps)	C_{abs} (μM)	C_{molec} (mM)
**UV/Vis TA**	360	0.1	63 (58)	3 (6)
**TRIR**	400	0.2	93.2 (93.2)	6 (17)
**TCSPC**	344	300	0.039 (0.039)	1.6 (4.2)

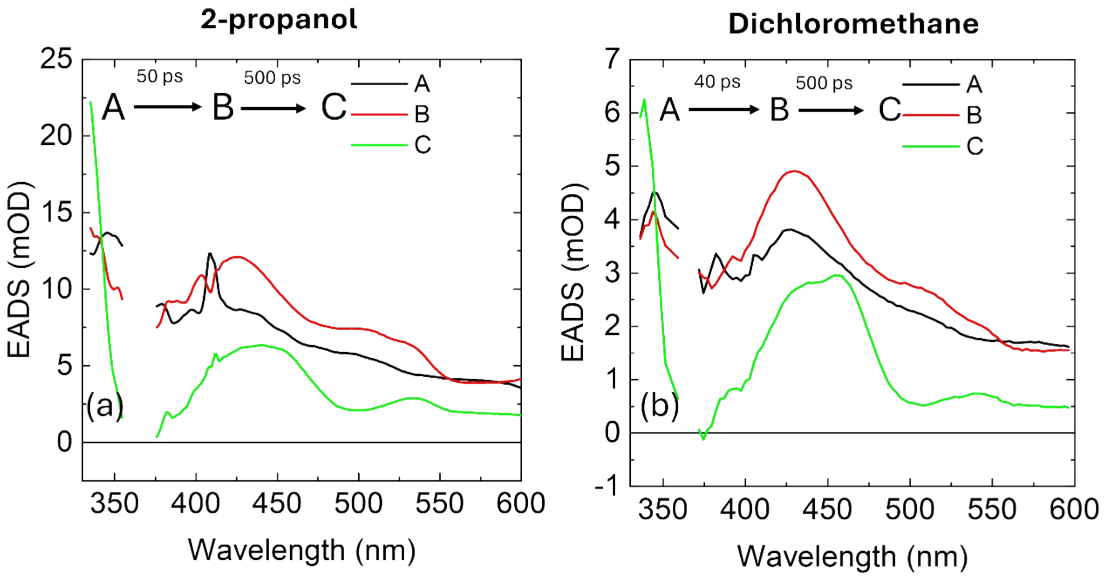

Fig. S1. Comparison of BAPO UV/Vis TA dynamics in 2-propanol (a) vs. DCM (b).

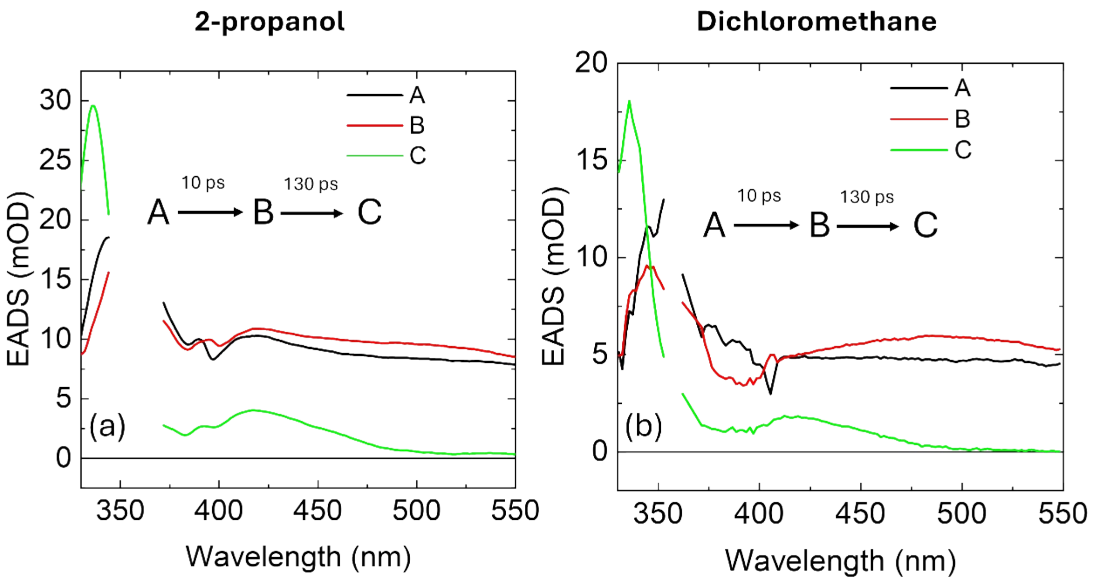

Fig. S2. Comparison of TPO UV/Vis TA dynamics in 2-propanol (a) vs. DCM (b).

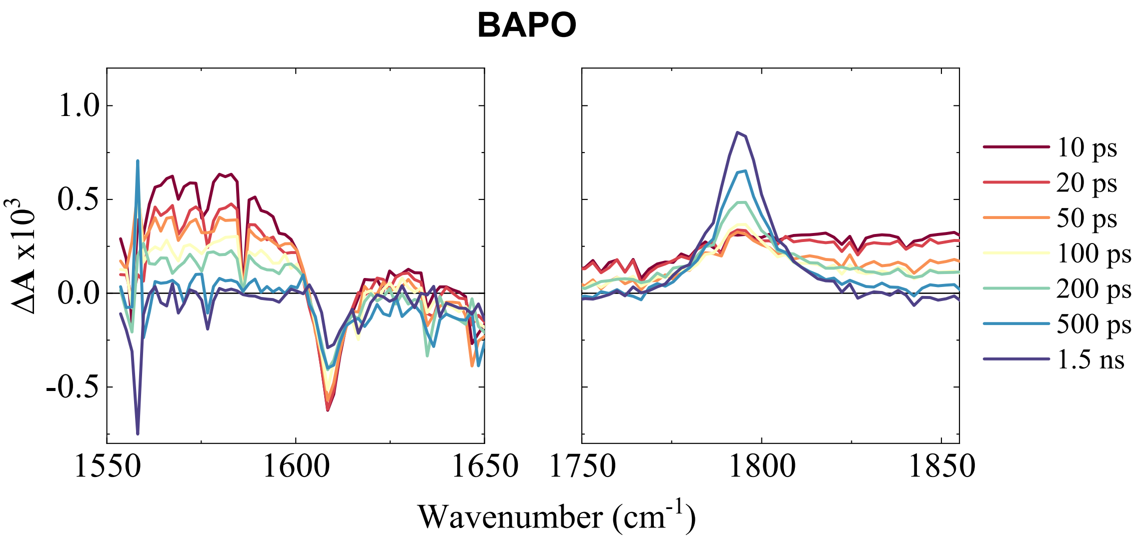

Fig. S3. Experimental TRIR spectra of BAPO following 320 nm excitation in DCM.

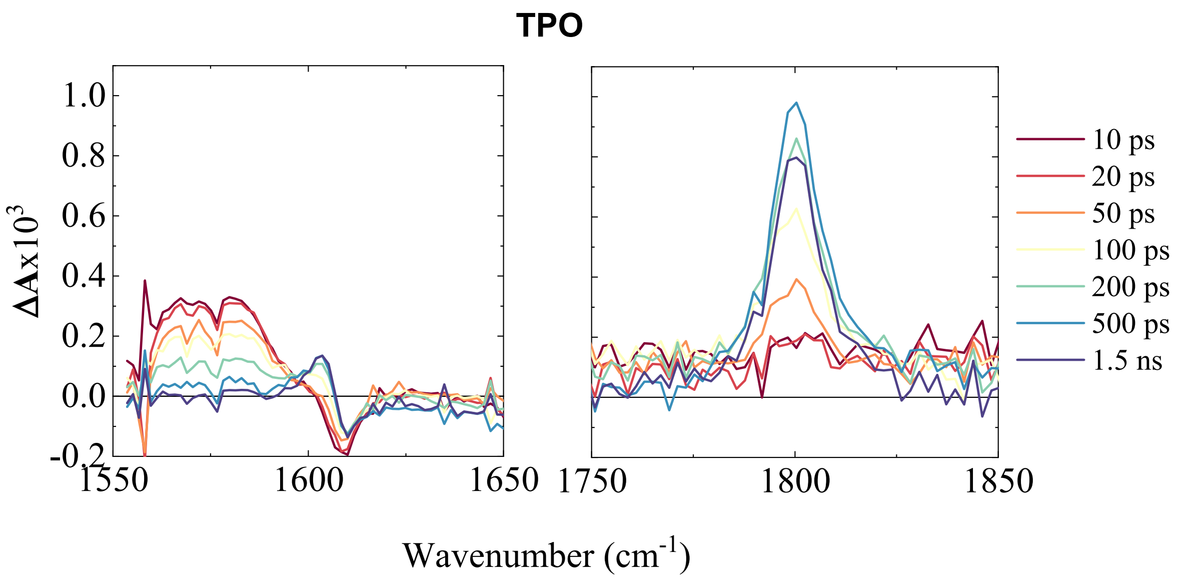

Fig. S4. Experimental TRIR spectra of TPO following 320 nm excitation in DCM.

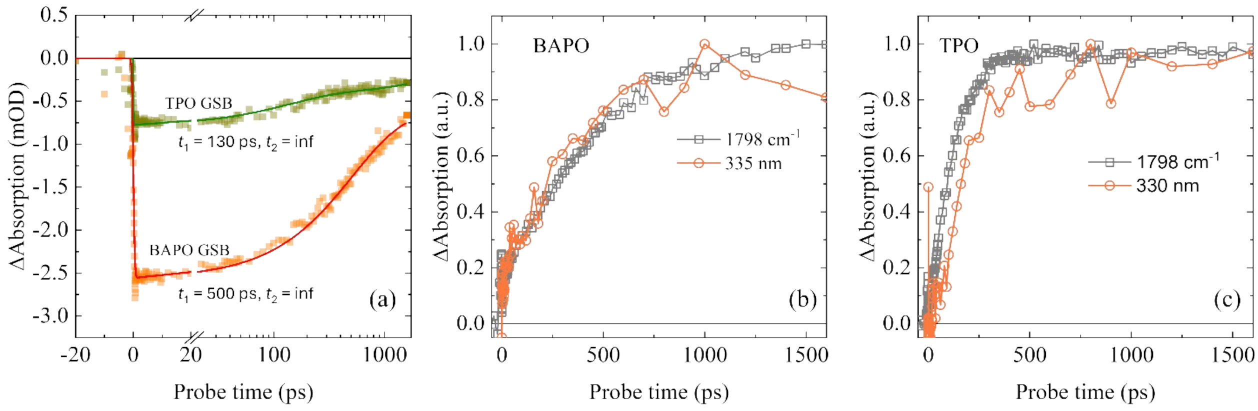

Fig. S5. (a) Exponential fit of BAPO and TPO ground-state bleach kinetics measured during fs-MIR experiments. Panels (b) and (c) show the comparison between the kinetic traces from UV-VIS (2-propanol) and fs-TRIR experiments (DCM). The initial TA signals from UV-VIS kinetic traces were subtracted and the kinetic traces then were normalized to their maximum signals.

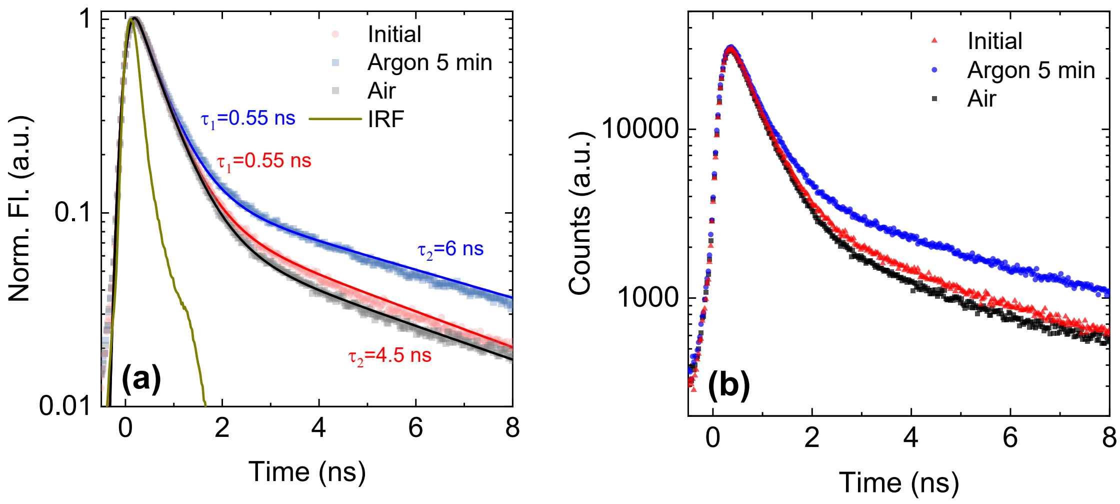

Fig. S6. Fluorescence decay traces of BAPO isopropanol solution, measured at 520 nm, before (red trace) and after (blue trace) purging with argon for 5 minutes. The grey trace indicates the recovered original trace obtained after the argon-purged solution was flushed with ambient air for 5 minutes. All traces are normalized to their maximum values for comparison. The relative amplitudes slightly increase from 10% to 13% when the solution is purged with argon. The panel (b) shows the raw non-normalized traces.

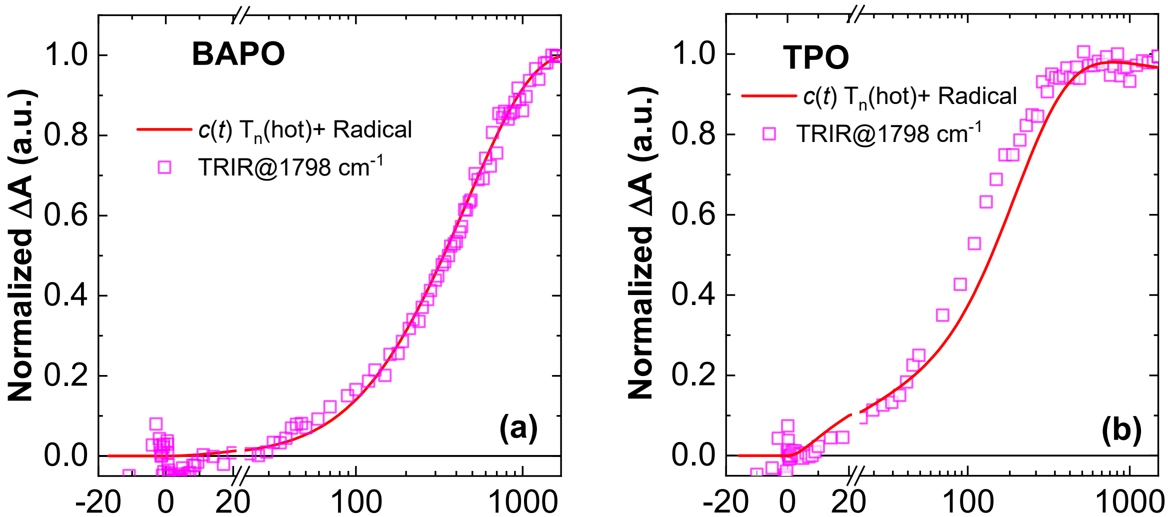

Fig. S7. Comparison of a superposition of species temporal evolution (**T_n(hot) + Radicals**) obtained by target analysis of UV/Vis TA data and experimental Mes-CO radical IR ESA growth for (a) BAPO and (b) TPO. The solid lines represent the concentration plots *c(t)* of T_n(hot) and Radical species superposition, whilst the hollow squares indicate the experimental TRIR kinetic traces at 1798 cm^{-1} frequency. The initial TA signals in both cases were subtracted from the traces.

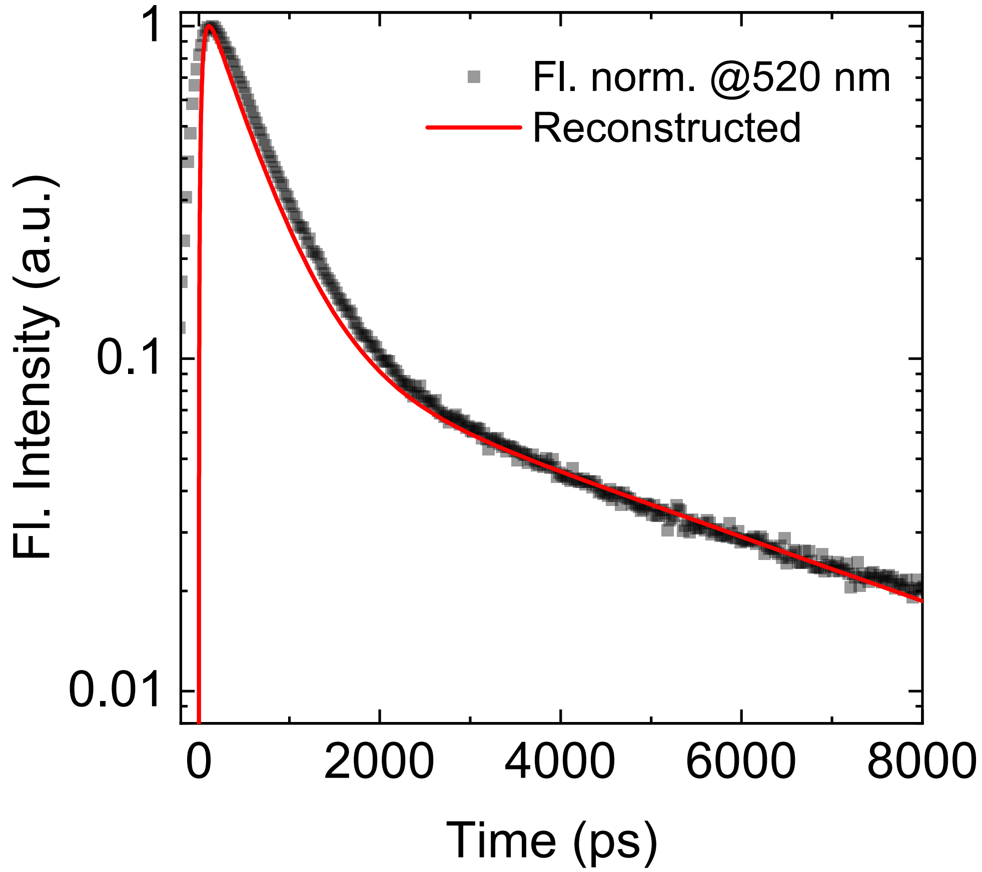

Fig. S8. Experimental (black) and reconstructed (red) TCSPC emission decay at 520 nm by superposition of concentration temporal evolution 1.65***S_1 cold** +0.1*(**T_n hot+T_1 cold**) of UV/Vis TA species.
